# The “canine first technique” in maxillary impacted canines: analysis of the treatment duration and success of therapy

**DOI:** 10.3389/froh.2024.1444018

**Published:** 2024-08-22

**Authors:** Stefania Perrotta, Tecla Bocchino, Massimo Amato, Ambrosina Michelotti, Vittorio Simeon, Vincenzo D’Antò, Pasquale Piombino, Emanuele Carraturo, Stefano Vollaro, Rosa Valletta

**Affiliations:** ^1^ Division of Orthodontics, Department of Neurosciences, Reproductive Sciences and Oral Sciences, University of Naples “Federico II’, Naples, Italy; ^2^ Department of Medicine and Surgery, University of Salerno, Salerno, Italy; ^3^ Department of Mental Health and Preventive Medicine, Medical Statistics Unit, University of Campania “Luigi Vanvitelli”, Naples, Italy; ^4^ Division of Maxillofacial Surgery, Department of Neurosciences, Reproductive Sciences and Oral Sciences, University of Naples “Federico II’, Naples, Italy

**Keywords:** impacted canine, surgical exposure, canine first technique, canine eruption, oral health

## Abstract

**Objectives:**

The goal of the study was to analyze the eruption time of the maxillary impacted canines treated with the “canine first technique” and evaluate the success rate.

**Materials and methods:**

A total of 103 patients with 131 impacted canines were treated. Alpha angle, Erickson–Kurol sectors, and age were studied to assess the difficulty of canine eruption. All the canines were treated with the “canine first” approach. The median follow-up time was evaluated using the Kaplan–Meier inverse procedure. The primary outcome (canine eruption time) was analyzed using Kaplan–Meier curves. The curve comparison between the different known risk factors was made using the log-rank test. The median eruption time (95% confidence interval) was calculated for each result.

**Results:**

The majority of the canines (66.4%) were placed palatally and positioned in sector 3 (30.16%). The median alpha angle was 38.7°. In 88.9% of cases, canines erupted and the median time of eruption was 4.2 months. A statistically significant difference in alpha angle >/<22° able to influence the eruption time was assessed. The variation of the alpha angle (>/<22°) has found to be statistically significative when compared to the eruption time variation.

**Conclusion:**

The canine first technique is effective for the eruption of impacted canines, and an alpha angle <22° can be considered a favorable prognostic factor.

## Introduction

Canine teeth play a vital role in ensuring overall dental esthetics, providing lip support, facilitating proper masticatory function, and contributing to disarticulation during lateral movements (1–3). The correct eruption, position, and morphology of canines within dental arches are crucial for maintaining oral health. Impacted canines pose a significant challenge in orthodontic practice, and early diagnosis and treatment are essential for achieving optimal results (4).

Certain clinical signs can indicate the presence of an impacted canine, such as the persistence of deciduous canines beyond 14–15 years of age, the absence of a canine bulge or its palatal presence, and deviations in the normal tip of the lateral incisors (5, 6). In addition, radiographic examinations are crucial for an accurate diagnosis (7). In 1988, Ericson and Kurol proposed the use of three geometric parameters detectable on panoramic radiographs: alpha angle, sectors, and distance from the occlusal plane (8).

**Figure 1:**
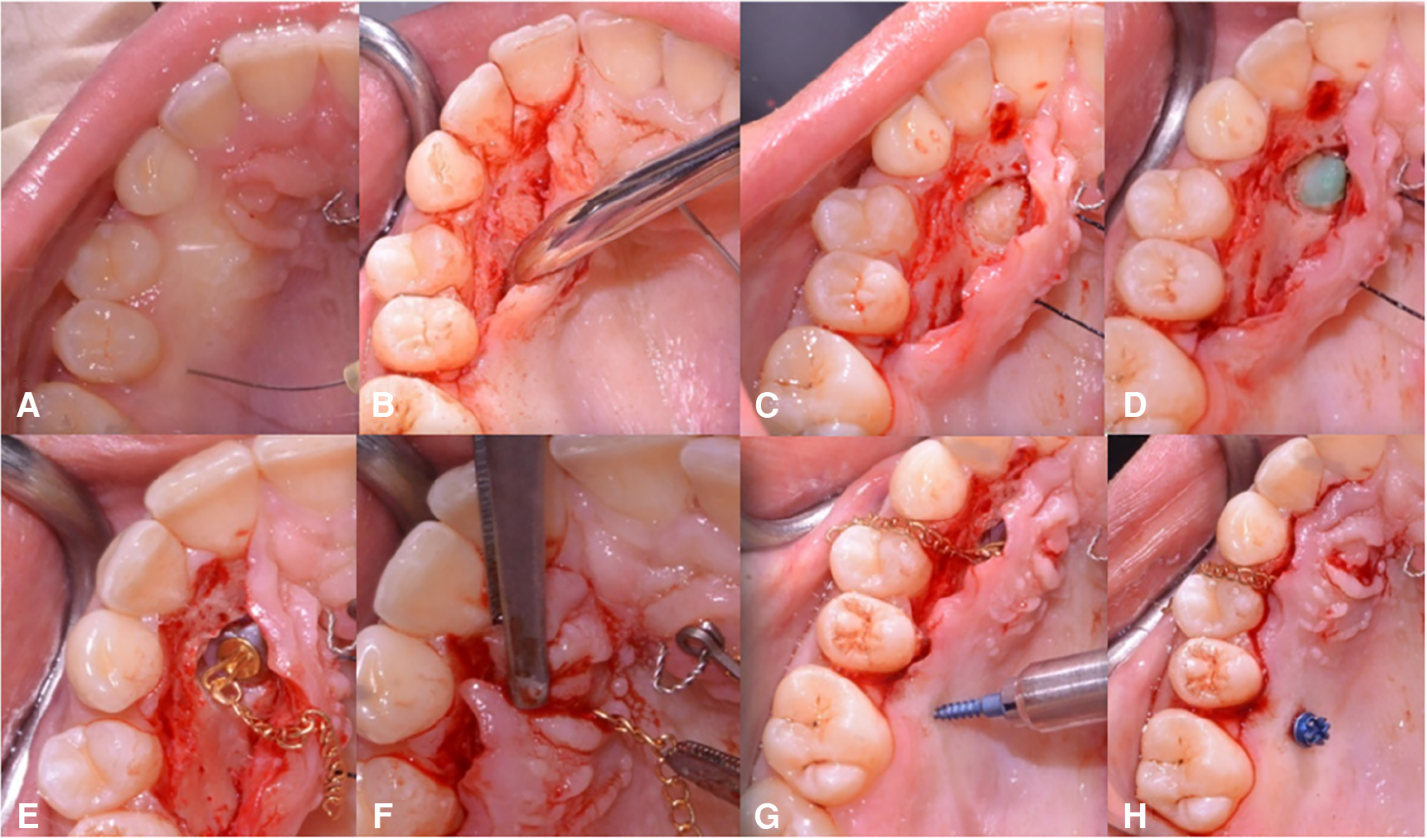
Description of the “canine first technique”. After local anesthetic infiltration, a full-thickness mucoperiosteal flap is performed **(A–D)**. A wire chain is then bonded to the crown **(E,F)**. Next, orthodontic traction of the canines is performed, exclusively using a beta-titanium cantilever (0.019 × 0.025) inserted into the 10 mm mini-screw in a palatal safe zone without a multibracketed orthodontic appliance **(G,H)**. Reprinted with permission from Bocchino et al. (9).

Several therapeutic strategies have been developed to restore the proper canine position within the dental arch. The “canine first technique” (Figure 1) involves a surgical approach to impacted canines before initiating fixed orthodontic treatment (9, 10). This combined surgical-orthodontic procedure aims to address palatal impacted canines. The goal of this study was to evaluate the eruption time of maxillary impacted canines treated with the “canine first technique” and assess the success rate of canine eruption in both younger and older populations.

## Materials and methods

The retrospective monocentric study was conducted at the Orthodontics and Gnathology Operative Unit, Federico II University Hospital, Naples, Italy. The cohort was enrolled between 2014 and 2019. A total of 103 medical records and orthopantomographies of 103 patients, treated in the same Operative Unit, were available for the study.

The inclusion criteria were:

• unilateral or bilateral palatally displaced canines treated with surgical exposure and planned for orthodontic alignment;
• a canine cusp tip position (documented by panoramic radiographs) from the long axis of the lateral incisor greater or equal to sector 2 (8, 11);
• an alpha angle >10°; and
• age from 13 to 36 years.

There were 131 impacted canines, of which 6 were mandibular and therefore excluded from the study. The following conditions were considered as additional exclusion criteria:

• craniofacial anomalies;
• congenital syndromes of craniofacial interest;
• periodontal disease; and
• temporomandibular disorders.

The study was carried out on radiographic and clinical evaluations of patients currently and no longer in treatment. The patients underwent impacted canine surgery through the “canine first” approach.

The sample was divided as follows:

• By age into two groups: under 16 and over 16 years of age.
• By gender into two groups: female and male.
• By alpha angle into two groups: >22° and <22°.

### Primary outcome measures

The samples thus collected were studied and divided by age, waiting times, and the success rate of canine recovery. The median time of observation was 14.1 months. The recovery times of the impacted canines were determined from the data present in the clinical records of the patients. The dates of surgery and the first post-surgical check in which the canine crown was visible in the arch were taken into consideration to find the primary outcomes, the former as the date of entry into the study and the latter was considered for the final calculation. The time of the eruption was measured in days/months from the day of surgery to the eruption or the last clinical control.

### Secondary outcome measures

The success ratio was determined by calculating the percentage of visible canine crowns in the oral cavity after treatment. To interpret the available clinical data, we considered a success as number 1 and a failure as 0. The alpha angle and sector position of the cusp were measured using panoramic radiographs. Previous panoramic radiographs, obtained from clinical records, were analyzed to evaluate the position of the impacted canine. Matte acetate tracing paper and a 0.3 mm HB fine pencil were used to trace the radiographs, and a protractor was used to measure all the parameters. The midline was determined as the line passing between the anterior nasal spine and the alveolar process. In this study, the canine sectors considered were from sector 2. The occlusal plane was defined by a horizontal line passing through the central incisors and the cusp of the first permanent molar. If the central incisor or first molar was absent, the lateral incisor and second permanent molar were used as references. In cases of mixed dentition, the horizontal line between the central incisors margin and the second molar was considered as the occlusal plane. Each radiograph was evaluated for the alpha angle and the Ericson–Kurol sector corresponding to the position of the impacted canine cusp. The analysis of these data and radiographs was performed by two experienced orthodontists. The information collected from the orthodontic folders was divided as follows:

#### Before starting treatment

- General anamnesis: name, sex, and date of birth.
- Medical anamnesis: relevant diseases, hospital admissions, drug use, allergies, pregnancy status, and smoking status.
- Panoramic x-ray of dental arches.
- Date of surgery.

After 12 months or at the last check date:

- Date of the canine eruption (if present).

#### Digital workflow

**Figure 2:**
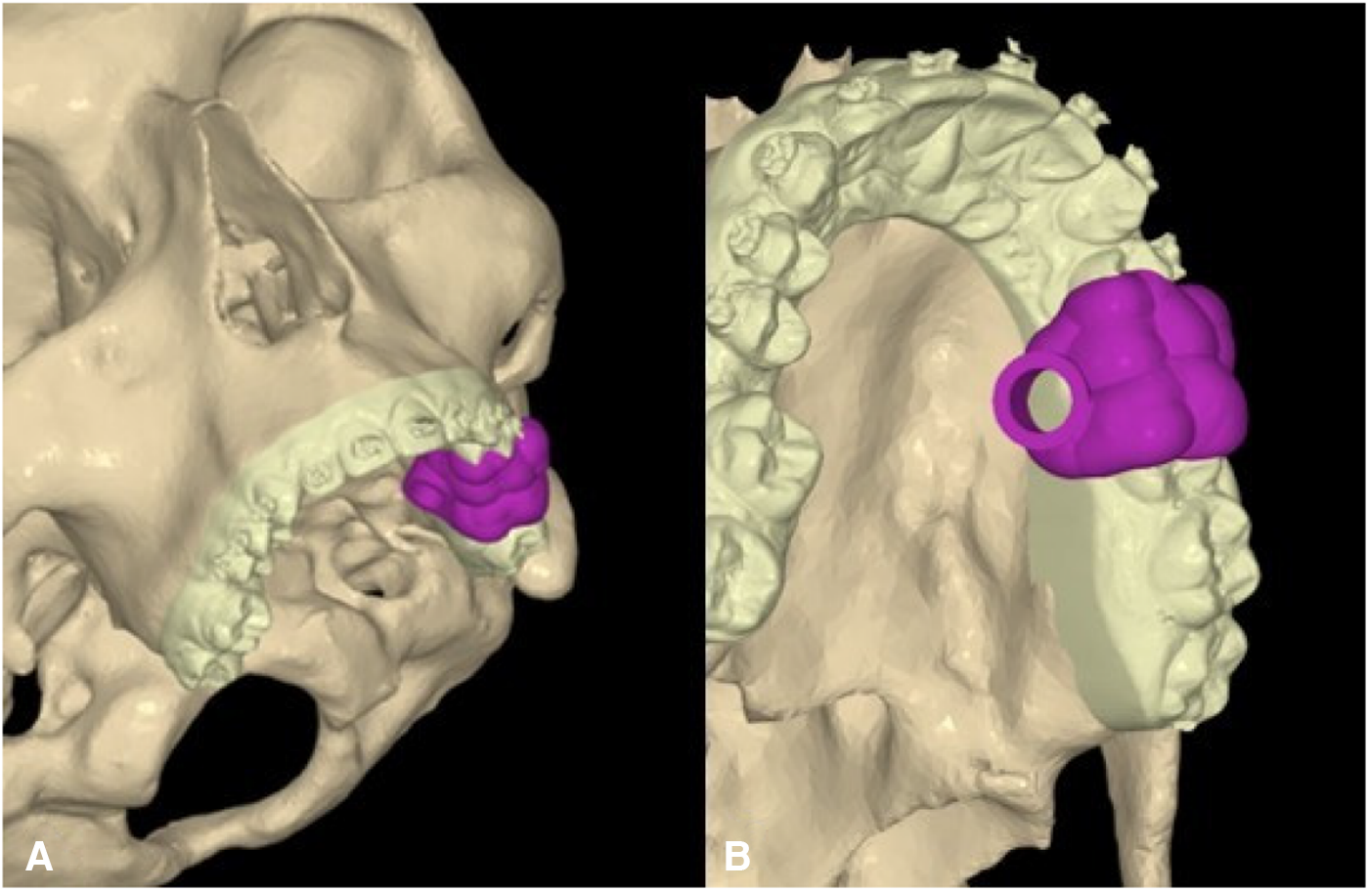
Virtual planning of CAD models for screw insertion. **(A)** Frontal view. **(B)** Occlusal view.

DICOM files from Cone Beam CT of the jaws were all taken from the same CT Scanner (Planmeca ProMax 3D, Helsinki, Finland). STL files of dentures and occlusion were taken with a three-dimensional dental scan (iTero Element 5D, Align Technology, Inc., Tempe, AZ, USA). Solid models of the mini-screws, teeth, and bone were then imported and elaborated using NEMOfab software (NEMOTEC SL, Madrid, Spain) to segment and generate computer-aided design (CAD) models based on the screw insertion direction at the infrazygomatic zone and palatal inter-radicular zones. In addition, a matching was created with STL files derived from the intraoral scans to digitally create a surgical guide useful for the correct insertion of mini-screws through the meshmix function offered by NEMOfab software (Figures 2A,B).

### Statistical analysis

The variables of interest were initially analyzed using descriptive statistics. Continuous variables were presented as either mean and standard deviation (SD) or median and range, depending on the distribution of the data. Categorical variables were presented as absolute and relative frequencies. The median follow-up time was evaluated using the inverse Kaplan–Meier procedure.

The primary outcome, canine eruption, was analyzed using Kaplan–Meier curves, and 95% confidence intervals (CIs) were calculated. A comparative analysis of the curves among different known risk factors was performed as an exploratory analysis using the log-rank test. For each result, the median eruption time was calculated along with its corresponding 95% confidence interval.

## Results

A total of 60 women and 43 men underwent treatment for the disinclusion of their impacted canines. The average age of the patients was approximately 18.2 years, with an SD of 5.7 years. The median age was 16 years.

The most frequent impacted tooth was the 13 with a prevalence of 54.2%, followed by the 23, which was impacted in 41.2% of the cases analyzed. In 66.41% of the patients, the canine was placed palatally, and in 29.8% of patients, it was located vestibularly. The patients were almost equally distributed across the five Erikson–Kurol sectors, with the highest proportion of patients falling into sector 3 (30.16%). Regarding the alpha angle, 125 observations with a mean size of 38.7° (SD of 15.19) were observed. Therefore, the success of the eruption of the impacted canines was analyzed, with successful eruptions occurring in 88.5% of patients. In addition, the median time needed for the canine eruption in at least 50% of patients was considered (Table 1).

**Table 1:** Percentage of patients for impacted tooth, position, alpha angle, sectors, and age.

Tooth	13	23	33	43	
	54.2%	41.2%	1.5%	3.1%	
Position	V	P	L	Cc	
	29.8%	66.4%	0.8%	3%	
Alpha angle	p25	P50	P75		
	29°	38°	46°		
Sector	1	2	3	4	5
	9.5%	14.3%	30.2%	27.8%	18.2%
Age	>16 years	<16 years			
	65.5%	50.5%			

The time elapsed from the surgery to the eruption was 4.2 months, with a 95% confidence interval between 3.4 and 5.3, considering all the samples. These data provided an opportunity to investigate whether the eruption time could vary depending on whether some factors were considered. The first factor examined was age: the target of 16 years was set, and the variation of the eruption time was evaluated by dividing the sample between less and more than 16 years of age.

The time elapsed in this cohort was 2.85 months, with a 95% confidence interval between 1.83 and 5.08. In the case of patients aged over 16 years, the median time elapsed was 5 months, with a 95% confidence interval between 3.8 and 6.09. The log-rank test was used to check whether the two curves were statistically significantly different and there was no detectable difference (*p* = 0.07). There was not enough evidence to determine whether the two cohorts, over 16 and under 16 years, were statistically significantly different in terms of eruption time (Table 2).

**Table 2:** Variation in eruption time based on age.

Age	Patients	50%	Standard error	95% CI
<16	67	2.85	0.72	1.83–5.08
>16	64	5	0.58	3.8–6.09
Total	131	4.2	0.52	3.44–5.27

The second factor analyzed was the alpha angle. The sample was divided into two groups: the former with an alpha angle greater than 22° and the latter with an alpha angle less than 22°. A statistically significant difference in the alpha angle was found. The 15 subjects with an alpha angle less than 22° had a median time of eruption of the impacted canine of 2.75 months, with a 95% confidence interval from 0.459 to 3.44, and for the 110 subjects who had an alpha angle greater than 22°, the time was 4.29 months, with a 95% CI from 3.67 to 5.27. The log-rank test for equality of survivor functions confirmed the statistically significant difference (*p* = 0.015) (Table 3).

**Table 3:** Patients divided by the alpha angle.

Alpha angle	Patients	50%	Standard error	95% CI
<22°	15	2.75	0.44	0.45–3.44
>22°	110	4.29	0.47	3.67–5.27
Total	125	4.13	0.51	3.4–5.11

The third factor considered to have a potentially significant influence on the eruption time was the sector and oral position of the impacted canines. Initially, sector 1 was excluded, and the median eruption time was 4.13 months, with a 95% confidence interval ranging from 3.40 to 5.11 months. However, a statistically significant difference in eruption time was not obtained.

Next, the vestibular canines were examined, and a statistically significant difference in the eruption time was not found neither combining the eruption time of canines in sector 1 and in vestibular position.

When focusing on palatal canines, no statistically significant difference in eruption time was found. Finally, the eruption times of canines in sector 2 were compared with those in the macro sector 3–4–5. The canines of sector 2 erupted after 2.85 months, with a 95% confidence interval from 1.83 to 5.67, unlike those located in sectors 3, 4, and 5, which showed a median eruption time of 4.09 months, with a 95% CI between 3.04 and 5.08. The log-rank test showed that this difference was not statistically significant (*p* = 0.29) (Table 4). The eruption times of the individual sectors were then merged into a single table and, considering the median and relative confidence intervals for the individual groups, it was concluded that the median eruption time was 4.09 months, with a 95% CI from 3.21 to 5.08 (Table 5).

**Table 4:** Patients divided by sectors.

Sectors	Patients	50%	Standard error	95% CI
1–2	18	2.85	0.69	1.83–5.67
3–4–5	96	4.09	0.40	3.04–5.08
Total	114	4	0.33	3.04–4.98

**Table 5:** Eruption time based on single sectors.

Sector	Patients	50%	Standard error	95% CI
1	12	5.50	2.78	1.37–11.96
2	18	2.85	0.69	1.83–5.67
3	38	2.98	0.65	1.37–4.88
4	35	5.27	1.04	3.04–8.26
5	23	4.13	0.54	2.52–5.08
Total	126	4.09	0.51	3.21–5.08

Fisher's test was used to evaluate the rate of success associated with the variation in sector, position, alpha angle, and age. The statistically significant difference was found only when combining the chart of the eruption rate that considers the alpha angle with the evaluation previously performed with the already statistically significant variation in the alpha angle less than or greater than 22°. In conclusion, we must note that a skeletal anchorage device [temporary anchorage device (TAD)] was used in 103 subjects (78.63%).

## Discussion

A total of 60 women and 43 men with impacted canines were treated in this study, which was consistent with the literature showing a higher frequency of impacted canines in women (58.2% vs. 41.8%). The average age of the patients was approximately 18.2 years, with an SD of 5.7 years and a median age of 16 years.

The focus was evaluating the time of eruption of the impacted canines, which represents a novel approach compared with previous studies that primarily assessed the duration of the entire treatment process, including eruption and alignment in the arch (9–15). The median eruption time for all 131 treated canines using our technique was 4.196 months. Unlike Becker and Chaushu, who measured eruption time based on the number of visits, we calculated it based on the months elapsed from the day of surgery (16).

In addition to eruption time, we evaluated various clinical and radiographic characteristics of the subjects. Our findings revealed that 58.25% of the cases were women, and the impacted canines more frequently observed were 13 and 23, with a higher prevalence of 13 than 23 (54.20% vs. 44.22%). Of the total impacted canines, 87 were located palatally and 39 vestibularly, which was consistent with existing literature (17, 18).

Regarding radiographic analysis, a mean alpha angle of 38.75° and a slight prevalence of sector 3–4–5 was found. The variation in the alpha angle, specifically being greater or less than 22°, was the only variable that significantly affected the eruption time. This finding aligns with the importance of the alpha angle as a prognostic factor, as indicated by Ericson and Kurol. However, we did not observe statistically significant differences in eruption time between sector 2 and the other sectors, contrary to the suggestions by Ericson and Kurol (8, 19, 20).

The success rate of the treatment was 88.85%, with a total of 116 out of 131 impacted canines successfully recovered in the dental arches. It should be noted that the eruption of some canines may have occurred after the date of the last check-up visit recorded in the medical records.

Regarding the surgical approach, either an open or closed technique or apical positioned flap (APF) was chosen, based on the individual patient's needs. However, the tunnel technique was not employed due to negative results in terms of impacted canine recovery (21). The use of TADs was prevalent in our cases (78.63% vs. 21.37%), as they provide skeletal anchorage and help mitigate adverse effects (22, 23).

Type IV titanium mini-implants were chosen and ensured a minimum insertion torque of 35 N, as calculated by a dynamometer. Surgery was performed through ultrasonic. Piezosurgery is now becoming increasingly popular in oral surgery and dentistry. The use of low frequency ultrasonic microvibrations offers precise and safe osteotomies in periodontology, overcoming the limitations of traditional instruments and increasing intraoperative control and safety in difficult anatomical zones. It is a minimally invasive technique due to its selective cutting of mineralized tissues, improved visibility, and decreased blood loss (24, 25).

In this study, a CAD-computer-aided manufacturing method was used to design surgical guides for the placement of mini-screws. The virtual planning method made it possible to precisely elaborate the insertion position of the screws at the infrazygomatic level, thus avoiding the roots of dental elements and other noble structures. Through an in-house method, the surgical guides were then printed using stereolithographic resins. Kivovics et al. has already demonstrated the usefulness of virtual surgical planning in the treatment of impacted canines. In their series, they compared the procedures with the help of pre-surgical virtual planning and with the traditional way without any CAD planning. Surgery was deemed successful in all patients in both groups. During healing, no complications were observed. The duration of surgery decreased significantly in the test group (4 min 45.1 s ± 1 min 8.4 s) compared with that in the control group (7 min 22.3 s ± 56.02 s) (26). Although other studies have also previously evaluated the usefulness of virtual surgical programming in the treatment of impacted canines, to date, only Camps-Font and Vilarrasa have shown a method for the design and implementation of surgical guidance for the placement of mini-screws for the treatment of palatally impacted canines (27). In our study, the production of a custom-made in-house surgical guide ensured a reduction in complications, surgical time, and convalescence time, at a very low total cost, according to the methods shown. The limitations of this method lie in the need to have a dedicated three-dimensional hub in one's own hospital/university department, as well as dedicated engineering or healthcare personnel familiar with the use of such software, the learning curves of which are not always rapid. On the other hand, in the absence of such an organization, it is still possible to turn to dedicated three-dimensional printing companies in the area.

## Conclusion

The results of this study show that a decrease in the alpha angle has a statistically significant impact on reducing the eruption time of impacted canines. To further validate these findings, it would be beneficial to extend the study to a larger sample size and assess whether other examined parameters are also affected by the reduced sample size. As the Operative Unit in which the study was conducted exclusively uses the “canine first” approach, it was not possible to compare the results with a control group. However, conducting a comparative study with other centers employing different techniques would be valuable in understanding the effectiveness of various approaches and comparing the outcomes.

## Data Availability

The original contributions presented in the study are included in the article/Supplementary Material, further inquiries can be directed to the corresponding author.
